
JOURNAL INFORMATION
==============================
NLM Title Abbreviation: ScientificWorldJournal
Journal ID: ScientificWorldJournal
NLM Journal ID: 101131163
Journal ID: TSWJ

The Scientific World Journal

ISSN: 2356-6140
EISSN: 1537-744X
Publisher: Wiley

ARTICLE INFORMATION
==============================
PMCID: PMC5956383
PMID: 15578119
DOI: 10.1100/tsw.2004.196
Article ID: 347807
Article version: 1
Subjects: Case Study

Renal Pelvis Squamous Cell Carcinoma and Renal Cell Carcinoma in a Tuberculous Kidney

Al-Assiri M. 1
Al-Otaibi M. F. 1
Sircar K. 2
Laplante M. 1
Department of Urology at Montreal General Hospital, McGill University, Montreal, Canada
2 Department of Pathology at Montreal General Hospital, McGill University, Montreal, Canada
Academic Editor: Anthony Atala

Publication date: 2004
Electronic publication date: 2004 Nov 18
Volume: 4
First page: 965
Last page: 968
Received 2004 Sep 5; Revised 2004 Oct 23; Accepted 2004 Oct 27
Copyright: Copyright © 2004 M. Al-Assiri et al.
Copyright year: 2004
License: This is an open access article distributed under the Creative Commons Attribution License, which permits unrestricted use, distribution, and reproduction in any medium, provided the original work is properly cited.
License URL: https://creativecommons.org/licenses/by/3.0/

Keywords: carcinoma, renal cell, squamous cell, tuberculosis, renal

==============================
Case Study
*TheScientificWorldJOURNAL* (2004) 4, 965–968
ISSN 1537-744X; DOI 10.1100/tsw.2004.196

### Renal Pelvis Squamous Cell Carcinoma and Renal Cell Carcinoma in a Tuberculous Kidney

M. Al-Assiri[1], M.F. Al-Otaibi[1], K. Sircar[2], and M. Laplante[1]
[1]*Department of Urology at Montreal General Hospital, McGill University, Montreal;*
[2]*Department of Pathology at Montreal General Hospital, McGill University, Montreal*

**E-mail**: manaalassiri@yahoo.com

*Received September 5, 2004; Revised October 23, 2004; Accepted October 27, 2004; Published November 18, 2004*

---

**KEYWORDS**: carcinoma, renal cell, squamous cell, tuberculosis, renal

**DOMAIN**: urology, pathology

---

### INTRODUCTION

Squamous cell carcinoma (SCC) of the renal pelvis is a rare entity, usually associated with chronic inflammation and/or infection. There have been several reports of SCC in tuberculous kidneys, however, to the best of our knowledge, we report the first case of a tuberculous kidney with both squamous and renal cell carcinoma (RCC) combined in one kidney.

### CASE

A 70-year-old male presented to the emergency room with left flank pain and fever. He was treated for renal tuberculosis 30 years prior to his presentation. Physical exam revealed left costoverteberal angle tenderness, while laboratory exam demonstrated leukocytosis and pyuria. Serum renal function tests were normal. He was initially treated as an acute pyelonephritis with intravenous antibiotics. A CT scan of the abdomen and pelvis showed an enhancing mass at the lower pole of the left kidney; further work-up shows no evidence of metastasis. He subsequently underwent a left radical nephrectomy with an uneventful postoperative course. Pathological findings were consistent with old renal tuberculosis (Fig. 1), SCC of renal pelvis (Fig. 2), and RCC (Fig. 3).

Patient has no evidence of metastasis after 3 years follow-up.

### DISCUSSION

Genitourinary tuberculosis occurs in approximately 15% of patients with pulmonary tuberculosis in developing countries[1]. SCC of the renal pelvis is a rare entity and represents less than 0.7–7% of renal pelvic tumors[2]. As with other sites in the genitourinary tract, SCC are usually secondary to chronic inflammatory processes, which is not surprising in a tuberculous kidney.

*Corresponding author.
©2004 with author.

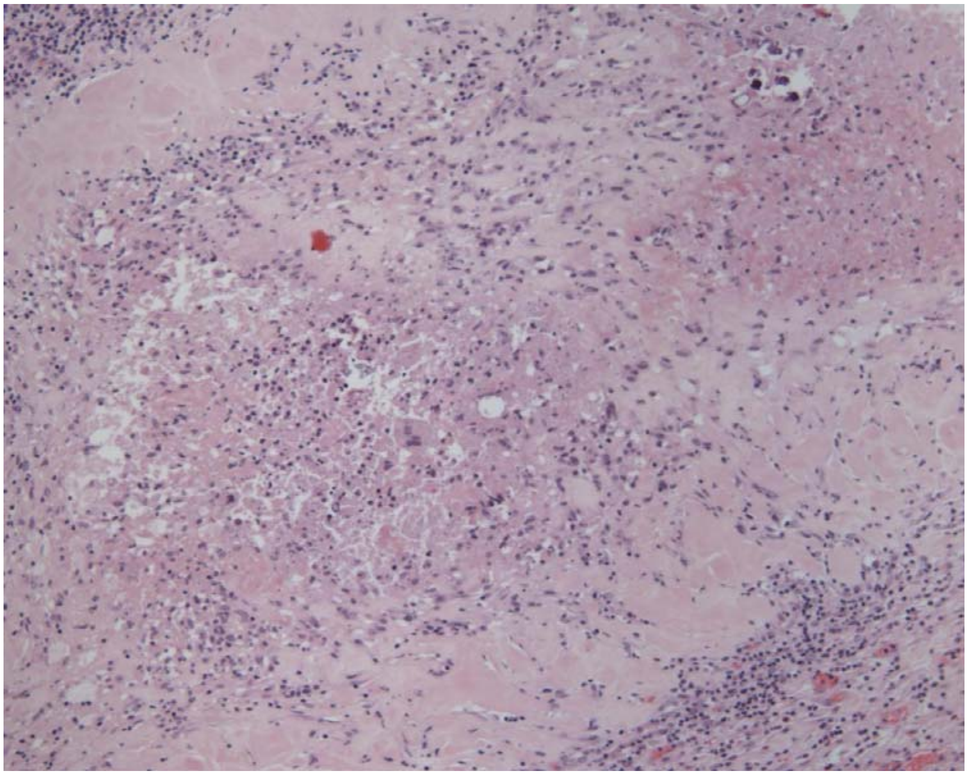

**FIGURE 1**

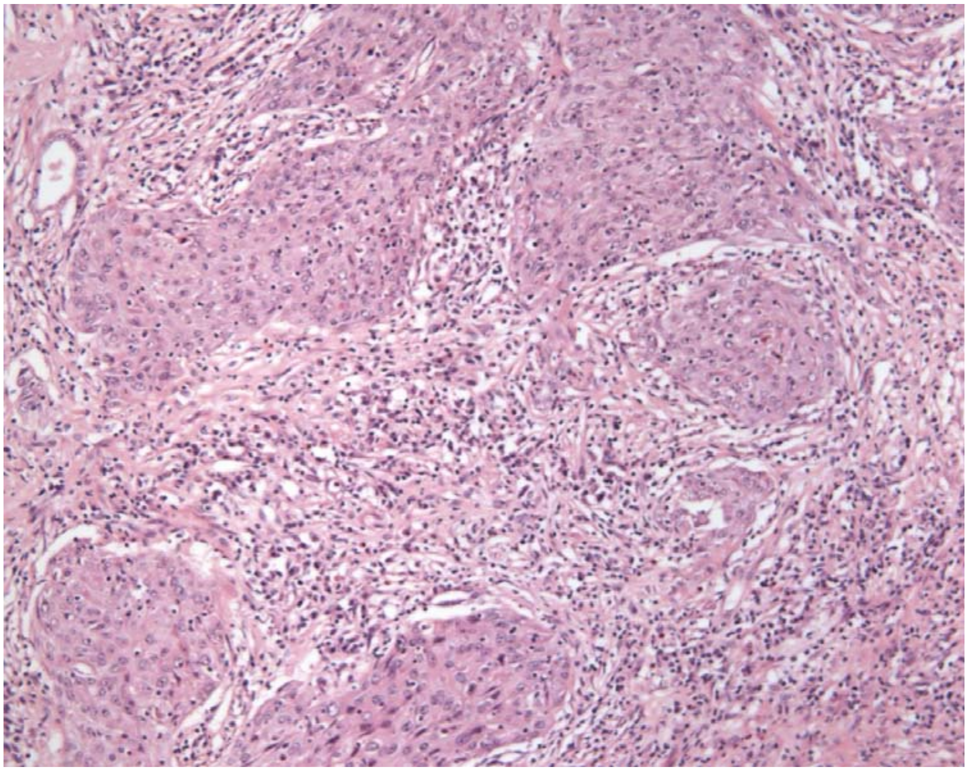

**FIGURE 2**

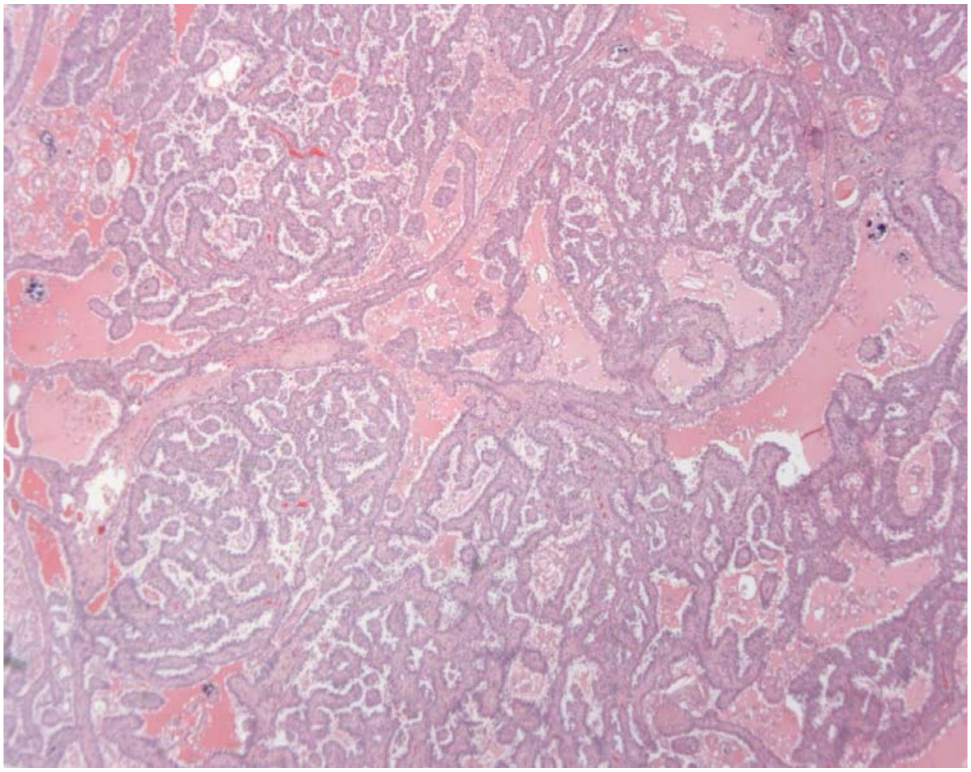

**FIGURE 3**

Preoperative recognition of SCC is very difficult since the symptoms will be obscured by stone presence. The presenting symptoms are abdominal pain, hematuria, constitutional symptoms, and palpable mass. Although SCC is not considered multifocal disease[3], a radical nephroureterctomy with bladder cuff excision is the preferred surgical intervention. Nephrectomy for renal tuberculosis is controversial, however, sepsis, pain, bleeding, hypertension, or suspected malignancies are considered indications for nephrectomy.

Approximately 45 cases of RCC in tuberculous kidneys have been reported in the literature. However, there have not been recommendations to follow these patients; this may be related to the rarity of tuberculosis in the west, which is not the case in other parts of the world like India and other developing countries in which tuberculosis is endemic.

Surveillance of GU tuberculosis with ultrasound of the kidney may be warranted to detect neoplastic pathology at early stages especially in those countries where tuberculosis is endemic. However, a pulmonary tuberculosis patient should not be screened routinely for GU tuberculosis unless the patient has clinical symptoms that are suggestive of GU tuberculosis.

The causative relationship of tuberculosis and RCC is rather complicated especially if we know from previous literature[4] that BCG interaction with uroepithelium upregulates interleukins and various interferons, which are the primary immunotherapy for RCC today.

### CONCLUSION

We report the first case in the world where these three pathologies coexist in one kidney.

### REFERENCES

1. Freedman, L.R. (1979) In *Strauss and Welt's Diseases of the Kidney*. 3rd ed. Earley L.E. and Gottschalk, C.W., Eds. Little, Brown, Boston. p. 859.
2. Babaian, R.J. and Johnson, D.E. (1980) Primary carcinoma of the ureter. *J. Urol*. **123**, 357.
3. Blacher, E.J., Johnson, D.E., Abdul-Karim, F.W., and Ayala, A.G. (1985) Squamous cell carcinoma of renal pelvis. *Urology* **25(2),** 124–126.
4. Watanabe, E., Matsuyama, H., Matsuda, K., Ohmi, C., Tei, Y., Yoshihiro, S., and Ohmoto, Y. (2003) Urinary interleukin-2 may predict clinical outcome of intravesical bacillus Calmette-Guerin immunotherapy for carcinoma in situ of the bladder**.** *Cancer Immunol. Immunother*. **52(8),** 481–486. Epub 2003 Apr 18.

**This article should be referenced as follows:**

Al-Assiri, M., Al-Otaibi, M.F., Sircar, K., and Laplante, M. (2004) Renal pelvis squamous cell carcinoma and renal cell carcinoma in a tuberculous kidney. *TheScientificWorldJOURNAL* **4**, 965–968.

**Handling Editor:**

Anthony Atala, Principal Editor for *Urology* and *Tissue Engineering*— domains of *TheScientificWorldJOURNAL*.
